# A Functional Beverage from Coffee and Olive Pomace: Polyphenol-Flavonoid Content, Antioxidant, Antihyperglycemic Properties, and Mouse Behavior

**DOI:** 10.3390/foods14081331

**Published:** 2025-04-11

**Authors:** Analy Aispuro-Pérez, Fernando Javier Pedraza-Leyva, Alicia Ochoa-Acosta, Mayra Arias-Gastélum, Feliznando Isidro Cárdenas-Torres, Bianca Anabel Amezquita-López, Emiliano Terán, Emmanuel Aispuro-Hernández, Miguel Ángel Martínez-Téllez, Roberto J. Avena-Bustillos, Selina C. Wang, Eli Terán-Cabanillas, Ulises Osuna-Martínez

**Affiliations:** 1 Facultad de Ciencias Quimico Biologicas, Universidad Autonoma de Sinaloa, Ciudad Universitaria, Culiacan 80013, Sinaloa, Mexico; est.analyap@uas.edu.mx (A.A.-P.); bamezquita@uas.edu.mx (B.A.A.-L.); 2 Facultad de Ciencias de la Nutricion y Gastronomia, Universidad Autonoma de Sinaloa, Avenida Cedros y Calle Sauces S/N, Culiacan 80019, Sinaloa, Mexico; fernando.pedraza@uas.edu.mx (F.J.P.-L.); daochoa@uas.edu.mx (A.O.-A.); mayra.arias@uas.edu.mx (M.A.-G.); feliznando@uas.edu.mx (F.I.C.-T.); 3 Facultad de Ciencias Físico-Matemáticas, Universidad Autónoma de Sinaloa, Ciudad Universitaria, Culiacan 80013, Sinaloa, Mexico; eteran@uas.edu.mx; 4 Centro de Investigacion en Alimentacion y Desarrollo, A.C., Carretera Gustavo Enrique Astiazaran Rosas 46, Hermosillo 83304, Sonora, Mexico; eaispuro@ciad.mx (E.A.-H.); norawa@ciad.mx (M.Á.M.-T.); 5 Western Regional Research Center, US Department of Agriculture, Agricultural Research Service, 800 Buchanan Street, Albany, CA 94710, USA; roberto.avena@usda.gov; 6 Department of Food Science and Technology, University of California, Davis, CA 95616, USA; scwang@ucdavis.edu

**Keywords:** amylase, antioxidant, biological activity, coffee, olive pomace, flavonoid, phenols

## Abstract

Background: Coffee is widely consumed worldwide and is rich in polyphenols with antioxidant properties linked to a reduced risk of metabolic and cardiovascular diseases. Olive pomace (OP), a by-product of olive oil production, contains phenolic compounds with cardioprotective effects but is often discarded. Combining it with coffee could enhance health benefits and promote sustainability. Methods: Soluble solids, total phenols, flavonoids, and antioxidant capacity (DPPH^•^ scavenging activity) were analyzed in C-OP at 5%, 10%, 15%, and 20% OP concentrations. The C-OP 10% brew was selected for further evaluation with α-amylase inhibition and a 14-day pilot study in a murine model, evaluating weight, food and liquid intake, and behavior, compared to a control group. Results: Adding OP powder to ground coffee increased the total phenol content in the brews. The highest antioxidant activity (6.62–8.17 mmol TE/L) was found in those brewed from 10%, 15%, and 20% concentrations. The C-OP 10% brew had the highest acceptance in mice, with increased consumption, greater exploratory behavior, and reduced resting time. It also showed 30.5% α-amylase inhibition at 200 µg/mL. Conclusions: The incorporation of OP into coffee enhances its total phenol content and antioxidant capacity. The C-OP 10% brew showed optimal bioactivity, suggesting its potential as a functional beverage for metabolic health.

## 1. Introduction

Coffee is among the most widely consumed beverages globally, with daily consumption surpassing two billion cups [1]. Its appeal extends beyond its rich flavor and stimulating effects to its complex chemical composition, particularly its high concentration of bioactive compounds such as polyphenols [2,3]. These compounds are renowned for their antioxidant properties, which enable them to neutralize free radicals and protect the body from oxidative damage [4]. Studies have reported that moderate coffee consumption is associated with a lower risk of neurodegenerative diseases such as Alzheimer’s and Parkinson’s, as well as cardiovascular conditions like hypertension, and metabolic disorders, including obesity and type 2 diabetes mellitus (T2DM) [4,5,6,7,8]. Among its primary phenolic compounds, Chlorogenic acids and their derivatives stand out due to their potent antioxidant activity, anti-inflammatory properties, and ability to modulate glucose metabolism [9].

In addition to its antioxidant capacity, coffee exerts multiple biological effects that support cardiometabolic health [10]. While it may temporarily raise blood pressure, it does not increase long-term hypertension risk [10]. Evidence suggests that it may reduce the risk of metabolic syndrome [11] and non-alcoholic fatty liver disease [12]. Additionally, its consumption follows a U-shaped pattern in lowering all-cause mortality, meaning that moderate intake provides the greatest benefits, while very low or excessive consumption reduce this effect. These effects define coffee as a functional beverage with potential longevity benefits [10,11,12,13,14,15].

Likewise, olive pomace (OP)—a by-product of olive oil production—has gained growing attention in recent years due to its abundance of phenolic compounds, including hydroxytyrosol, oleuropein, and tyrosol [3]. Despite its rich content of bioactive compounds, OP is often discarded, posing significant environmental challenges [16]. These compounds have been extensively studied for their antioxidant, anti-inflammatory, cardioprotective, and antihyperglycemic effects [17,18,19,20,21], which are key benefits associated with olive oil consumption in the context of the Mediterranean diet [18,19]. Additionally, evidence suggests that OP compounds contribute to metabolic health by improving glucose tolerance and insulin resistance. These effects are mediated through the modulation of key metabolic pathways involved in chronic inflammation, lipid metabolism, and mitochondrial function. Studies have shown that OP polyphenols can inhibit α-glucosidase and α-amylase activity, leading to a reduction in postprandial glucose levels, while also enhancing insulin sensitivity by regulating signaling pathways such as AMPK and PPAR-γ [22,23,24].

Beyond metabolic regulation, OP has demonstrated neuroprotective properties, with certain polyphenols exhibiting the ability to reduce neuroinflammation, protect against amyloid aggregation, and enhance synaptic function, suggesting a potential role in neurodegenerative disease prevention [25]. Given these multifaceted biological activities, OP emerges as a promising functional ingredient with potential applications in disease prevention and the development of functional foods and sustainable bioactive formulations.

The combination of coffee and OP presents a promising opportunity for developing functional products, as both ingredients are rich in polyphenols with well-documented antioxidant- and health-promoting activities. Coffee polyphenols, such as Chlorogenic acids and caffeine, have been shown to strongly inhibit lipid oxidation inhibition and energy metabolism enhancement properties. Meanwhile, OP polyphenols, particularly hydroxytyrosol, exhibit remarkable anti-inflammatory and hepatoprotective effects. The synergistic interaction of these ingredients may amplify their individual health benefits, potentially reducing the risk of chronic diseases, including neurodegenerative, cardiovascular, and metabolic disorders [7,8,20,21].

Beyond their health benefits, the valorization of agro-industrial by-products aligns with sustainability goals and circular economy principles [16]. Repurposing OP not only adds economic value to the olive oil production chain, but also mitigates its environmental impact by reducing waste generation. Moreover, incorporating functional ingredients into coffee—a widely consumed beverage—may facilitate market adoption and increase consumer access to health-enhancing compounds [16].

Addressing chronic diseases and oxidative stress necessitates innovative dietary strategies. The development of functional foods enriched with bioactive compounds offers a viable approach to improving both public health and environmental sustainability. In this context, the present study evaluates a novel coffee and olive pomace (C-OP) brew, assessing its soluble solids, total phenolic and flavonoid content, antioxidant capacity, α-amylase inhibitory activity, and consumer acceptability in mice.

## 2. Materials and Methods

### 2.1. Extraction and Drying of OP

The separation of broken pits and skins and drying of OP paste were performed following the methodology established by Inzunza-Soto et al. [3]. Fresh Arbequina OP was obtained directly from the olive mill centrifuges at the California Olive Ranch during the 2023 olive harvest and transported to the Western Regional Research Center to be processed within three hours after collection at its Food Processing pilot plant. The separation of the broken olive skins and pits was conducted using a Langsenkamp pilot plant fruit pulper, model 150 (Warner Bodies, Elwood, IN, USA). The separation process was performed in two stages, as follows: first, using a stainless-steel sieve with 1.52 mm diameter perforations, and then with another stainless-steel sieve with 0.68 mm diameter perforations. The de-pitted OP paste was dried in a Buflovak atmospheric drum dryer (Hebeler Process Solutions, Tonawanda, NY, USA), with a 0.23–0.25 mm gap between the drums, operating at a temperature of 135 °C.

The pitted drum-dried OP flakes were milled in a Retsch ultracentrifugal mill type ZM 200 (Retsch GmbH, 42781 Haan, Retsch-Allee 1-5, Germany) fitted with a stainless steel 0.5 mm conical hole ring sieve to produce powders with less than 500 µm particle size. The dried OP powder was packaged in metalized bags with nitrogen flushing for oxygen removal, hermetically heat-sealed, and stored at 2 °C until being used in this study.

### 2.2. Determination of C-OP Blend Brew Concentrations

To evaluate the effect of the combination of coffee and olive pomace (C-OP), different brews were prepared in specific percentages of both ingredients. The concentrations were selected based on the sensory acceptance of a panel of individuals with previous studies conducted by our working group. In a preliminary investigation of the acceptance of commercial coffee blends at concentrations of 5%, 10%, 15%, and 20% of C-OP brews, it was observed that the blend with 10% OP was the most accepted by the participants. A pilot study has even been conducted in male Wistar rats comparing 10% and 20% C-OP blends, reporting a preference for the brew with 10% OP.

The concentrations of the brews are detailed as follows: blend 1: 100 g of coffee and 0 g of OP (100:0); blend 2: 0 g of coffee and 100 g of OP; blend 3: 95 g of coffee and 5 g of OP (5%); blend 4: 90 g of coffee and 10 g of OP (10%); blend 5: 85 g of coffee and 15 g of OP (15%); and blend 6: 80 g of coffee and 20 g of OP (20%); moreover, these brews were extracted in 177 mL of water per 10 g of dry base of C-OP (Table 1).

### 2.3. Extraction of C-OP Brewing

A commercial coffee maker (T-fal, Heliora Petit, model CM321DMX; Miguel de Cervantes Saavedra No. 169, Piso 9, Colonia Granada, Miguel Hidalgo, Mexico City, CDMX, Mexico) was used with an ultra-fine filter No. 4 (No. 4 coffee filters, model 46-1541801HT; Ringstr. 99, 32427 Minden, Germany). Before starting the extraction, the selected filter was moistened, and the mixture of ingredients was placed inside. For this, a commercial 100% Arabica Colombian coffee with 500 µm particle size (ground medium) and medium-dark roast was used.

The coffee and OP blends were brewed following the manufacturer’s instructions. The process was repeated two more times to obtain a triplicate.

### 2.4. Determination of Soluble Solids

The evaluation of soluble solids of coffee, OP, and C-OP extract blends was performed using a digital refractometer ATAGO (501 Omaeda, Fukaya-shi, Saitama 369-1246, Japan) Model PAL-1. A triplicate 2.5 mL sample was placed in the refractometer, and the results were recorded. The results were expressed as g per 100 mL.

### 2.5. Determination of Total Soluble Phenols

The determination of total soluble phenols of coffee, OP, and C-OP extract blends was performed using the Folin–Ciocalteu method [26]. Briefly, 50 µL of the samples was mixed with 125 µL of 1 N Folin–Ciocalteu reagent and 3.125 mL of distilled water. The mixture was thoroughly homogenized and incubated for 5 min at room temperature (25 ± 2 °C). Subsequently, 750 µL of a 20% (*w*/*v*) sodium carbonate solution and 950 µL of distilled water were added, followed by vigorous stirring to ensure complete homogenization of the reagents. The solution was incubated in darkness at room temperature for 30 min. Absorbance was measured at 765 nm. The results were expressed as mg of Chlorogenic acid equivalents (CAE) per mL.

### 2.6. Determination of Total Flavonoids

The total flavonoid content was determined using the method described by Loarca-Piña et al. [26]. Briefly, 100 µL of the samples was mixed with 75 µL of a 5% NaNO_2_ solution. After 6 min, 150 µL of a 10% AlCl_3_ solution was added, and the samples were allowed to stand for an additional 5 min. Then, 0.5 mL of 1 M NaOH was added, and distilled water was added up to a total volume of 2.5 mL. The solution was thoroughly mixed, and the absorbance was measured against a blank prepared at 510 nm. The results were expressed as mg of (+)-catechin equivalents (CE) per mL.

### 2.7. Determination of Antioxidant Activity

The antioxidant activity of coffee and C-OP brews was evaluated using the 2,2-diphenyl-1-picrylhydrazyl (DPPH^•^) free radical scavenging method [27]. Briefly, a DPPH^•^ solution was prepared at a concentration of 6 × 10^−5^ mol/L in methanol. Then, 3.9 mL of this solution was mixed with 0.1 mL of the samples. The mixture was incubated in darkness at room temperature (25 ± 2 °C) for 30 min. Absorbance was measured at 515 nm and the % of radical inhibition was calculated. A Trolox standard curve was used to calculate the Trolox equivalent (TE), and antioxidant activity expressed as mmol TE/L.

### 2.8. Mouse Behavior Affected by C-OP Consumption

C57BL/6 mice (8 weeks old, with an initial weight of 30 ± 1 g) were acquired from BIOINVERT (Mexico City, Mexico). The animals were housed in a temperature-controlled room (25 ± 2 °C) under a 12-h light/12-h dark cycle, with free access to a standard diet (Lab Rodent Diet 5001) and water or the experimental beverage C-PO brew. Sixteen mice were divided into two groups—water or C-OP 10% brew consumption—and kept in individual acrylic boxes. This study was conducted following the principles and procedures described in the Mexican Official Standard NOM-062-ZOO-1999 [28]. Technical Specifications for the Production, Care, and Use of Laboratory Animals. The study protocol for the use of animals was approved by the Institutional Committee for Animal Use with number 2-02CB-2025 (Facultad de Ciencias Químico Biológicas, Universidad Autónoma de Sinaloa, Mexico).

**Figure 1:**
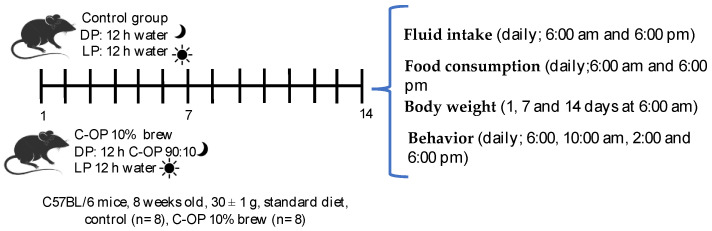
In vivo experimental design. C-OP: coffee and olive pomace.

The procedure established by Pereira-Alves et al. [29] and Kelley [30], with slight modifications, was followed. Food and liquid intake were measured every 12 h, at the beginning of each phase, as follows: light (inactive) phase (6:00 a.m.) and dark (active) phase (6:00 p.m.), the duration of phases was light (inactive) phase (from 6:00 a.m. to 6:00 p.m.) and dark (active) phase (from 6:00 p.m. to 6:00 a.m.) (Figure 1). The control group had access to water *ad libitum* during both phases, while the C-OP 10% brew group had access to the brews for the dark (active) phase (12 h) and water in the light (inactive) phase (12 h) for two weeks (14 days). Their body weight was recorded on days 1, 7, and 14 of the experiment at 6:00 a.m. Additionally, behavioral observations were conducted every 4 h (6:00 a.m., 10:00 a.m., 2:00 p.m., and 6:00 p.m.) throughout both phases (inactive and active). The evaluation included the presence and intensity (moderate, medium, and high) of active behavior (exploration and curiosity for stimuli), resting (sleep period), aggressiveness (greater tendency to attack when manipulated), anxiety (overreaction to stimulus), and stereotypies (repetitive movements).

### 2.9. Inhibitory Activity Against α-Amylase

The determination of α-amylase enzyme inhibition was performed according to Santos-Ballardo et al. [31]. In a 96-well microplate, 75 µL of α-amylase (AMY2) (0.5 mg/mL in 0.1 M phosphate buffer, pH 6.9) was mixed with 75 µL of the C-OP 10% brew (0.01, 0.1, 1, 10, 50, 100, and 200 µg/mL in 0.1 M phosphate buffer, pH 6.9) and incubated for 10 min at 37 °C. The negative control consisted of the reaction without an inhibitor (75 µL of 0.1 M phosphate buffer, pH 6.9), while acarbose was used as the positive control. Subsequently, 75 µL of starch (0.1% *w*/*v* in 0.1 M phosphate buffer, pH 6.9) was added and incubated for 10 min at 37 °C. Then, 125 µL of dinitrosalicylic acid (DNS, 96 mM in 0.05% sodium sulfite, 1% sodium hydroxide, and 0.2% phenol) was added, then heated in boiling water for 5 min. After cooling to room temperature, absorbance measurements were performed at 405 nm, and the results were expressed as % enzymatic inhibition obtained using the following formula:

(1) $$\%\;inhibition=\frac{Control\;Abs-Sample\;Abs}{Control\;Abs}\times 100$$

*Control Abs*: absorbance of the reaction without inhibitor.

*Sample Abs*: absorbance with inhibitor.

### 2.10. Statistical Analysis

Data analysis was performed using R statistical software (version 4.4.1; R Foundation for Statistical Computing, Vienna, Austria). A one-way ANOVA was performed to assess the effect of OP percentage on the measured parameters, including DPPH scavenging activity, total phenolics, flavonoids, and others. To improve the linear modeling of certain measured data, a logarithmic transformation was applied. Some figures present the transformed data, whereas their description in the text is provided in the original linear scale after applying the inverse transformation. Post hoc pairwise comparisons were performed using Tukey’s method to identify specific differences between infusions with different C-OP percentages. A *p*-value of ≤0.05 was considered statistically significant.

## 3. Results

### 3.1. Soluble Solids, Phenolics, Flavonoid Content, and Antioxidant Activity of Coffee, OP Brew, and C-OP Brews

Table 2 shows the concentrations of soluble solids, total phenols, and total flavonoids in coffee and OP brews. The OP brew exhibited a **94% higher** concentration of soluble solids than coffee (*p* < 0.05). Similarly, the total phenol content in the OP brew was **90% greater** than that in coffee (*p* < 0.001), highlighting its higher polyphenol contribution. In contrast, coffee’s total flavonoid content did not differ from that of the OP brew, although the latter showed **4.7% higher levels** (*p* > 0.05). Despite these total phenol content variations, no significant differences were observed in the free radical scavenging activity between the OP and coffee brews (*p* < 0.1), suggesting a comparable antioxidant capacity.

**Figure 2:**
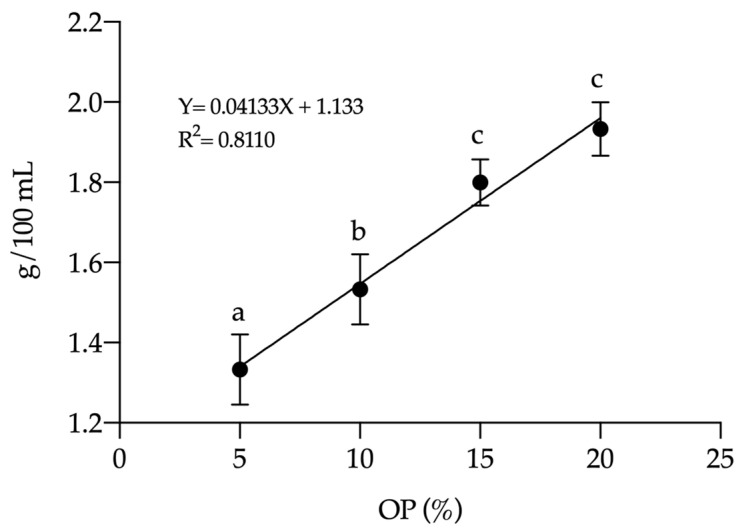
Soluble solid content of C-OP brews as a function of OP addition in coffee. Data points represent the mean values, while error bars indicate standard deviations. Different letters indicate statistical significance (*p* ≤ 0.05). OP: olive pomace.

The variation in soluble solid content is shown in Figure 2. A significant increase (*p* = 0.0249) was observed in the soluble solid concentration of the brews containing OP (5, 10, 15, and 20%), with increments of 25, 33.33, 50, and 58.33%, respectively, compared to the pure coffee brew. The relationship between OP concentration and soluble solids followed a linear trend, indicating that for every 10% increase in OP content, the concentration of soluble solids increased by approximately 0.41 g/100 mL (R^2^ = 0.81; *p* < 0.05). However, no significant differences (*p* = 0.62) were observed in the soluble solid concentration of the 15% (1.8 g/100 mL) and 20% (1.9 g/100 mL) brews compared to the OP brew (2.33 g/100 mL).

**Figure 3:**
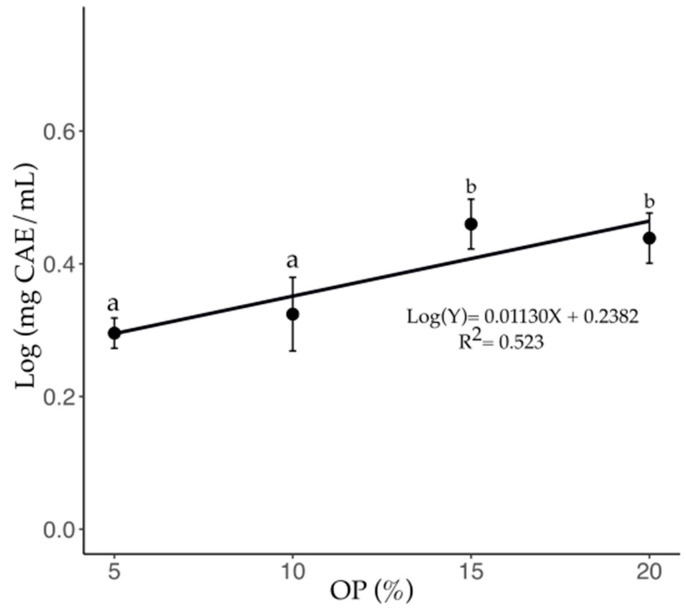
Total phenolic content of C-OP brews as a function of OP addition to coffee. Data points represent the mean values, while error bars indicate the standard deviation. Different letters indicate statistical significance (*p* ≤ 0.05). OP: olive pomace.

The variation in total phenol content in the C-OP brews is shown in Figure 3. A significant increase (*p* ≤ 0.05) was observed with the addition of OP at 5, 10, 15, and 20%, with increments of 90, 110, 190, and 170%, respectively, compared to the pure coffee brew (1.0 mg CAE/mL, Table 2). This equation (Log(Y)= 0.0113X + 0.23; R^2^ = 0.523; *p* < 0.0001) indicates that, as the OP concentration increases, phenol accumulation follows a nonlinear pattern, with a more pronounced increase at lower OP percentages and a tendency to stabilize at higher concentrations. No significant differences were observed between the 15% (2.9 mg CAE/mL) and 20% (2.7 mg CAE/mL) OP brews.

**Figure 4:**
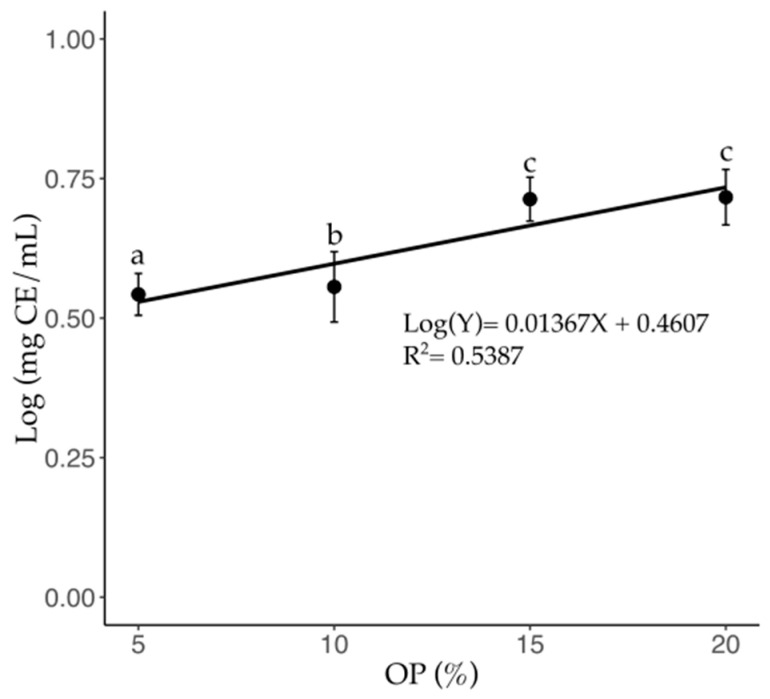
Total flavonoid content of C-OP brews as a function of OP addition in coffee. Data points represent the mean values, while error bars indicate the standard deviation. Different letters indicate statistical significance (*p* ≤ 0.05). OP: olive pomace.

The variation in flavonoid content is shown in Figure 4. A significant increase (*p* ≤ 0.05) was observed in the flavonoid concentration of the brews containing OP (5, 10, 15, and 20%), with increments of 2.78, 13.51, 28.38, and 44.44%, respectively, compared to the pure coffee brew (Table 2). This model indicates that for every 10% increase (R^2^ = 0.538; *p* < 0.0001) in OP content, flavonoid accumulation follows a nonlinear pattern, with a more pronounced rise at lower OP concentrations and a plateau at higher levels. Similar to the phenolic content, no significant differences were observed between the 15% (4.75 mg CE/mL) and 20% (5.2 mg CE/mL) OP brews.

**Figure 5:**
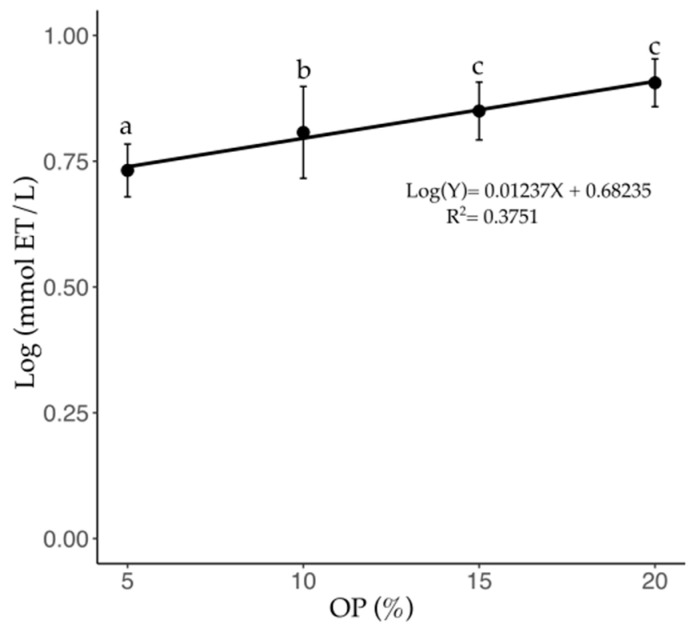
DPPH radical scavenging activity of C-OP brews. An indication of antioxidant activity as a function of OP addition in coffee. Data points represent the mean values, while error bars indicate the standard deviation. Different letters indicate statistical significance (*p* ≤ 0.05). OP: olive pomace.

The DPPH^•^ scavenging activity values in Figure 5 show significant differences (*p* < 0.05) as a function of the OP percentage in the C-OP brews, following concentration-dependent behavior. The brews enriched with 10, 15, and 20% OP presented log 5.60, 8.17, and 8.10 mmol TE/L, respectively, which corresponds to an increase of 20.0, 48.55, and 47.27%, respectivley, compared to that of the pure coffee brew (Table 2). The relationship between OP concentration and DPPH^•^ scavenging activity followed a logarithmic trend described by the equation indicating that, for every 10% increase in OP concentration, the DPPH^•^ scavenging activity increases by approximately 13.6% (R^2^ = 0.538; *p* < 0.0001).

### 3.2. Mouse Behavior Affected by C-OP Consumption

#### 3.2.1. Food Consumption

**Figure 6:**
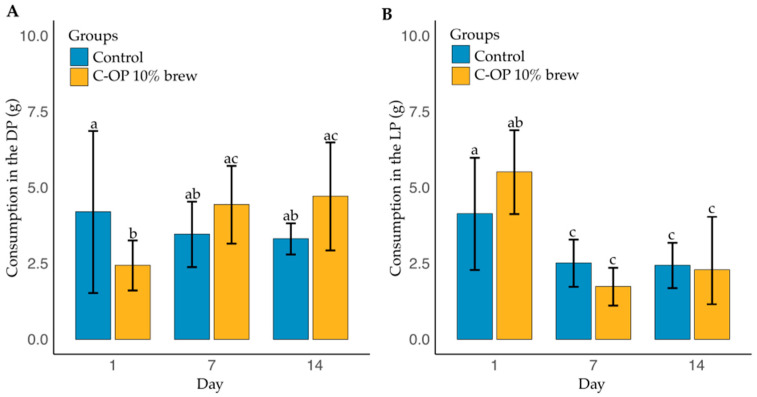
Food consumption in two different phases of C-OP 10% brew. (**A**) dark (active) phase: the blend group had a C-OP 10% brew, and the control group had water. (**B**) Light (inactive) phase: the blend group had a C-OP 10% brew, and the control group had water. The control group is represented in blue, while the C-OP brew group is shown in yellow. The bars indicate the mean food consumption (g) for each group per day, with error bars representing standard error. Different letters indicate statistical significance (*p* ≤ 0.05). C-OP: coffee and olive pomace; DP: dark (active) phase; LD: light (inactive) phase.

Figure 6A shows food intake during the dark (active) phase across the experimental groups. No significant main effects of group (*p* = 0.6462) or day (*p* = 0.3739) were observed, but a significant interaction (*p* = 0.0109) indicated variations across the days. The mean intake was 3.65 g in the control and 3.85 g in the C-OP 10% brew group, with no significant differences (*p* < 0.05). The largest difference occurred on day 1, where the control group consumed 1.76 g more than the C-OP 10% brew group (*p* = 0.2150). Intake in the C-OP 10% brew group tended to be lower on day 1 than on day 3 (*p* = 0.1160) and 14 (*p* = 0.0513).

Figure 6B displays food intake during the light (inactive) phase. No significant effect of group was found (*p* = 0.724), but food intake varied by day (*p* < 0.001). The control group had a mean of 3.02 g (median = 2.65 g), while the C-OP 10% brew group had a mean of 3.17 g (median = 2.90 g). No significant differences were found between the groups at any time point (*p* > 0.05), though differences were observed within the C-OP 10% brew group between the first and last days (*p* = 0.0105).

#### 3.2.2. Weight Gain

**Figure 7:**
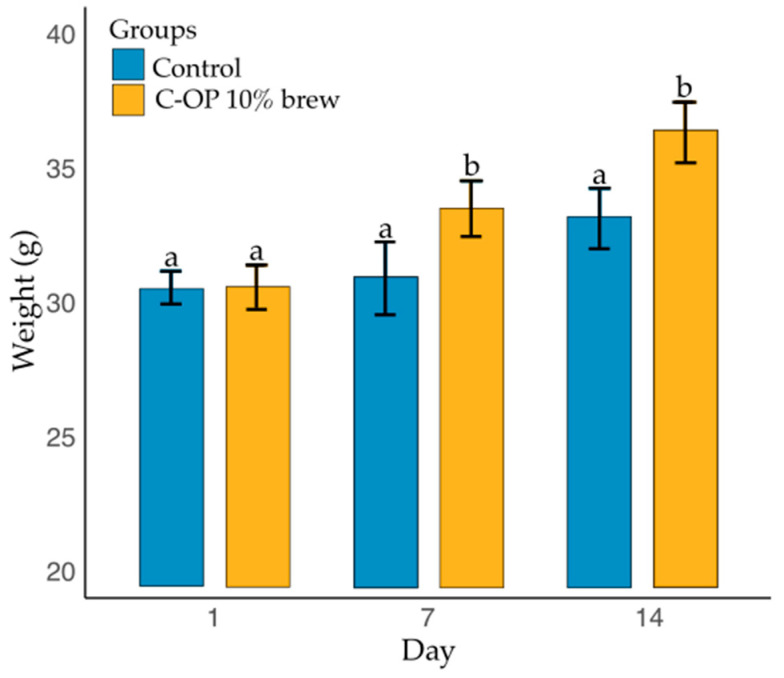
Weight gain of experimental groups. The control group is represented in blue, while the C-OP brew group is shown in yellow. The bars indicate the mean weight (g) for each group per day, with error bars representing the standard error. Different letters indicate statistical significance (*p* ≤ 0.05). C-OP: coffee and olive pomace.

Figure 7 illustrates the variation in mouse weight as a function of group and day. The analysis revealed a significant effect of both group (*p* = 0.001) and day (*p* < 0.0001) on body weight. However, the interaction between group and day was not statistically significant (*p* = 0.06).

The mice in the control group (coffee brew) had a mean weight of 31.5 g (median = 31.3 g), whereas those in the C-OP 10% brew group had a significantly higher mean weight of 33.5 g (median = 34). The mice in the C-OP 10% brew group, on day 14, had significantly higher body weight than those in the control group on the same day (*p* = 0.024). Additionally, the mice in the C-OP 10% brew group on days 7 and 14 had significantly greater weight than their control counterparts (*p* < 0.0001 and *p* = 0.0491, respectively). However, no significant differences were found between the groups on 1 day (*p* = 1.0000) or any other time points.

#### 3.2.3. Liquid Consumption

**Figure 8:**
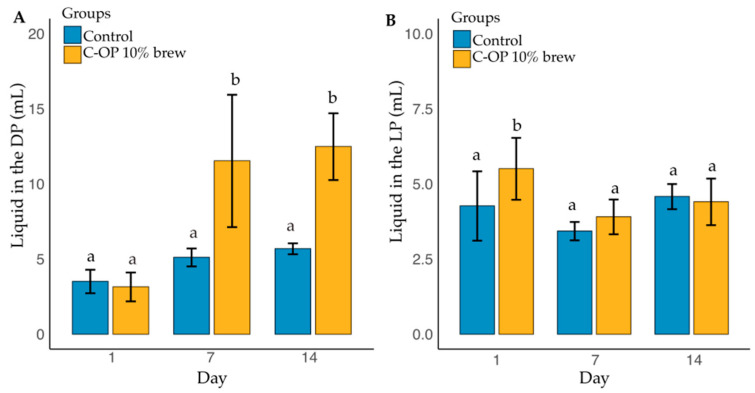
Liquid consumption in two different phases in the murine model. (**A**) Dark (active) phase: the blend group had a C-OP 10% brew, and the control group had water. (**B**) Light (inactive) phase: the blend group had a C-OP 10% brew, and the control group had water. The control group is represented in blue, while the C-OP brew group is shown in yellow. The bars indicate the mean liquid consumption (mL) for each group per day, with error bars representing standard deviation. Different letters indicate statistical significance (*p* ≤ 0.05). C-OP: coffee and olive pomace; DP: dark active phase; LD: light (inactive) phase.

Figure 8A shows liquid intake during the dark (active) phase across the C-OP 10% brew group. A significant effect was found for both C-OP 10% brew group (*p* = 0.016) and measurement day (*p* = 0.018), but no interaction was observed (*p* = 0.170). The control group with pure coffee brew consumed 4.76 mL, significantly less than the C-OP 10% brew group (9.05 mL, *p* = 0.045), reflecting a 90% increase. The control group (coffee brew) on day 1 had lower intake than the C-OP 10% brew group on day 14 (*p* = 0.04), and intake in the C-OP 10% brew group was lower on day 7 compared to day 14 (*p* = 0.03). No significant differences were found between groups on the same days (*p* > 0.05).

Figure 8B presents liquid intake during the light (inactive) phase. No significant effects of the C-OP 10% brew (*p* = 0.422), day (*p* = 0.286), or interaction (*p* = 0.662) were observed. The control group averaged 4.09 mL (median = 3.70), while the C-OP 10% brew group averaged 4.60 mL (median = 4.20). Despite slight numerical differences, no significant pairwise differences were found (*p* > 0.05).

#### 3.2.4. Mouse Behavior

The mice supplemented with the C-OP 10% brew (Table 3) exhibited high hyperactivity and reduced resting, whereas the control mice displayed typical behavior with moderate active behavior and longer resting periods.

During the dark (active) phase, no differences were observed on day 1. However, from day 7, and more prominently on day 14, the C-OP brew group showed increased activity and more active behavior, while the control mice maintained unchanged behavior.

In the light (inactive) phase, both groups exhibited similar behavior on day 1. From day 7 to 14, the C-OP 10% brew group showed increased active behavior (moderate to medium) and reduced resting (high to medium), whereas the control group remained unchanged throughout the study.

### 3.3. Inhibitory Activity Against α-Amylase

**Figure 9:**
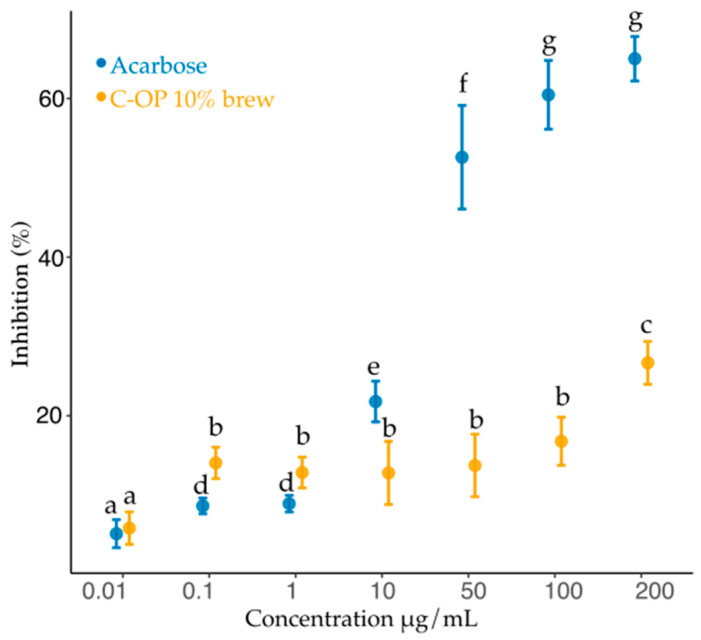
α-amylase enzyme inhibition of C-OP 10% brew. The inhibitory effect of acarbose is represented in blue, while the inhibitory effect of the C-OP 10% brew is shown in yellow. Data points represent mean values, with error bars indicating the standard error. Different letters indicate statistical significance (*p* ≤ 0.05), while similar letters indicate no significant difference (*p* > 0.05). OP: olive pomace.

The inhibition percentage of the C-OP 10% brew increased significantly with concentration (*p* < 0.001) (Figure 9). At 0.01 µg/mL, inhibition was 5.84%, rising to 14.1% at 0.1 µg/mL (*p* = 0.007). Concentrations from 1 to 50 µg/mL showed a similar trend (12.8–13.7%), while inhibition increased to 16.8% at 100 µg/mL (*p* = 0.001) and peaked at 26.7% at 200 µg/mL (*p* < 0.001), representing a 4.57-fold increase.

Acarbose, used as a positive control, exhibited inhibition values of 5.1, 8.6, 8.9, 21.8, 52.6, 60.5, and 65% at 0.01, 0.1, 1.0, 10, 50, 100, and 200 µg/mL, respectively.

Compared to acarbose, the C-OP 10% brew showed greater inhibition at 0.01, 0.1, and 1.0 µg/mL, with 12.1%, 39.0%, and 30.5% higher inhibition, respectively. However, at 10, 50, 100, and 200 µg/mL, its inhibition was lower, with acarbose surpassing it by 41.3%, 75.8%, 72.2%, and 58.9%.

## 4. Discussion

### 4.1. Composition of C-OP Brews

Coffee itself is a rich source of nutraceutical compounds, including Chlorogenic acids, flavonoids, xanthones, and caffeine, which exhibit potent antioxidant and anti-inflammatory properties. Among these, Chlorogenic acids play a key role in the health-promoting effects of coffee, contributing to glucose metabolism regulation, neuroprotection, and cardiovascular health [32].

The content of these bioactive compounds in brewed coffee varies according to the roasting degree and extraction procedure. Common brewing practices are not optimized for the complete extraction of bioactive components, but rather for the sensory properties of the brew, so it is important to evaluate the actual content of such bioactive compounds in obtained infusions. Although ground roasted coffee and dry OP have a similar dry matter content, their proximal and chemical composition differs significantly [33,34,35,36,37,38]. Our results show relevant differences in the soluble solids and total phenolic contents of coffee and OP brews. Higher contents of total phenolics have been recovered from OP water extracts [33] compared to ground coffee water extracts [35,36], which is in agreement with our findings, since soluble phenols may contribute to the total content of soluble solids in herbal infusions. Furthermore, differences between the total carbohydrate content in OP (66–85%) [34,35] and ground coffee beans (62–69%) [37,38] may also contribute to the observed linear increment in extracted soluble solids.

The incorporation of OP—a by-product of olive oil production—into coffee formulations enhances its bioactive profile by introducing additional polyphenolic compounds and other bioactive constituents. This combination not only enhances coffee’s functional properties, but also offers a sustainable approach to utilizing OP, which is often considered waste in the olive oil industry.

No previous studies have evaluated functional beverages made from coffee and OP, and, thus, the key characteristics, such as soluble solids, total phenols, and flavonoids, in these formulations remain unexplored. However, prior research has examined OP extracts obtained using different solvents. For instance, Zhao et al. [39] investigated aqueous, methanolic, and ethanolic OP extracts, reporting a phenolic content ranging from 36 to 43 mg CAE/g. Their findings highlighted that acidification significantly enhances total phenol extraction [39]. Other studies have explored the enrichment of coffee with bioactive compounds by incorporating dried plant powders. In a study by Rehab F. M. Ali [40], adding 0.25 to 1.25% *Mentha longifolia* to coffee increased the total phenol content from 3.7 to 14.88 mg GAE/100 g DW, while the total flavonoid content rose from 6.67 to 10.13 mg EQ/100 g DW. However, the study did not assess the impact on organoleptic properties [40]. Similarly, Latief et al. [41] examined the combination of *Coffea liberica* with 1, 3, and 5% dried *Curcuma zanthorrhiza*, observing a substantial increase in total phenol content from 1.04 to 6.87 mg GAE/g and flavonoid content from 9.89 to 16.23 mg QE/g compared to coffee alone, demonstrating a significant enhancement in bioactive compounds [41].

Unlike these previous studies, our work directly evaluated a coffee brew supplemented with OP, a by-product that some olive oil industries classify as waste [42,43]. Our findings demonstrated that OP addition increased not only the total phenol and flavonoid content, as observed in the studies by Rehab F. M. Ali [40] and Latief et al. [41], but also significantly raised the concentration of soluble solids in the beverage. This finding aligns with the well-documented bioactive potential of OP, which is rich in hydroxytyrosol and tyrosol—compounds known for their strong antioxidant properties and metabolic health benefits [32]. The observed increase in bioactive compounds underscores OP’s potential as a functional ingredient in coffee, reinforcing its role in promoting health benefits while contributing to the sustainable valorization of an olive oil industry by-product.

### 4.2. Content of Soluble Solids of C-OP Brews

The incorporation of OP into coffee influenced the total solid content, with linear increases of 35% to 45% as OP concentration rose. This effect is attributed to OP’s diverse composition, which includes phenolic compounds, fiber, fats, and other nutrients that contribute to the total mass of the brewed beverage [44].

The increase in total solids aligns with previous findings, where OP enhanced the bioactive profile of food products by increasing the presence of phenolic acids, flavonoids, and flavonoid polymers [45]. These compounds improve the nutritional value and may also influence sensory attributes, such as texture and mouthfeel, which are key to consumer acceptance [46]. The incorporation of OP into food matrices has been linked to improved product quality, suggesting its potential for enhancing functional beverages [47]. Interestingly, no significant differences were observed between the 10% and 15% OP brews, suggesting a saturation threshold. Beyond this point, additional OP does not further alter the total solid content, indicating that moderate OP incorporation is sufficient to achieve functional benefits without negatively impacting beverage consistency.

### 4.3. Content of Total Soluble Phenols of C-OP Brews

Coffee itself is a complex matrix rich in bioactive constituents, including Chlorogenic acids, particularly caffeoylquinic acids, the primary phenolic compounds linked to improved glucose metabolism, cardiovascular health, and antioxidant effects [48]. The addition of OP to coffee increased the total phenol content, with the highest levels observed in 15% and 20% OP brews. OP is a known source of phenolic compounds, such as hydroxytyrosol and tyrosol, which have strong antioxidant properties [39,40]. However, the 10% OP brew showed no significant difference compared to the 5% OP brew, suggesting a saturation effect or extraction limitations. Similarly, no differences were observed between the 15% and 20% OP brews, indicating a threshold beyond which additional OP does not further enhance phenol content [41]. This increase in total phenols has important implications for functional food development, improving both nutritional value and potential health benefits [42]. Studies have shown that incorporating bioactive ingredients into staple foods, such as tortillas enriched with avocado and cactus flours, can reduce cholesterol and glucose levels [43]. Likewise, OP supplementation in bread (10% and 20%) has been shown to improve nutritional profiles and increase polyphenol content [44].

Beyond antioxidant effects, OP may contribute to metabolic health, particularly in obesity management. Prior studies suggest that OP oil consumption reduces visceral fat and improves lipid profiles [46]. Additionally, OP bioactive compounds may help mitigate hyperglycemia in T2DM. Claro et al. [49] reported that OP oil enriched with triterpenic acids improved glucose tolerance and vascular function in obese mouse models, indicating a protective role against insulin resistance [49]. These findings reinforce OP’s potential as a functional coffee additive, enhancing polyphenol content while contributing to metabolic health. The next section examines its influence on soluble solids, further shaping the physicochemical and sensory attributes of C-OP brews.

### 4.4. Content of Total Healthy Flavonoids in C-OP Brews

The addition of OP to coffee brews resulted in a significant increase in flavonoid content; however, no substantial differences were observed between the 15% and 20% OP brews. This pattern mirrors the trend seen for total phenolic content, suggesting that flavonoid accumulation reaches a saturation point beyond an OP concentration of 15%. These findings align with previous studies that have demonstrated OP to be a rich source of bioactive compounds [50]. For instance, quercetin, a predominant flavonoid in OP, has been shown to exert beneficial metabolic effects. In diabetic mice, supplementation with 10 to 15 mg/kg of quercetin over 10 days led to reduced blood glucose and triglyceride levels, while simultaneously enhancing the activity of key metabolic enzymes such as hexokinase and glucokinase [51].

Moreover, flavonoids are known to activate the antioxidant pathways that contribute to their anti-inflammatory properties [52]. These compounds can inhibit enzymes such as lysozymes, reducing arachidonic acid release and thereby mitigating inflammatory reactions. Key flavonoids present in OP, including quercetin, genistein, apigenin, kaempferol, and epigallocatechin 3-gallate, have been shown to modulate the expression and activation of proinflammatory cytokines such as interleukin-1 beta (IL-1β), tumor necrosis factor alpha (TNF-α), interleukin-6 (IL-6), and interleukin-8 (IL-8), effectively regulating the expression of genes involved in inflammation [51,52].

Beyond their anti-inflammatory effects, flavonoids in OP have demonstrated potential in metabolic regulation, particularly through their ability to inhibit α-amylase activity—an enzyme essential for carbohydrate digestion [53]. This inhibition may play a crucial role in controlling postprandial blood glucose levels, offering potential benefits in the prevention and management of metabolic diseases such as T2DM [54]. These findings underscore the functional value of OP-enriched coffee brews, not only as a source of bioactive compounds, but also as a dietary strategy with implications for metabolic health.

### 4.5. Antioxidant Activity of C-OP Brews

The incorporation of OP into coffee brews led to a significant increase in antioxidant capacity, with brews containing higher OP percentages exhibiting approximately 50% greater activity. This enhancement underscores the role of OP in improving the antioxidant profile of food products. The observed increase in antioxidant activity is largely attributed to the elevated presence of antioxidant phenolics in the brewed samples, a relationship that has been well documented in previous studies [55,56]. Specifically, phenolic compounds such as hydroxytyrosol—recognized for its potent antioxidant properties—are likely contributors to these effects [56,57].

Interestingly, despite the numerical increase in antioxidant capacity, no differences were observed between brews containing 10%, 15%, and 20% OP. This finding suggests a saturation effect in the extraction process, where the mobile phase reaches a limit in its ability to solubilize additional bioactive compounds beyond a certain OP concentration [58]. This may indicate that, while moderate OP incorporation enhances antioxidant potential, excessive amounts may not yield further functional benefits.

Beyond their role in free radical scavenging, antioxidants from OP have been implicated in the regulation of cellular processes, particularly in mitigating oxidative stress—a key driver of chronic inflammation associated with metabolic disorders such as T2DM and obesity [50]. The capacity of these bioactive compounds to neutralize oxidative damage provides a mechanistic basis for OP potential health benefits.

Unlike previous studies that primarily focused on OP in dried powder or extract forms, our research evaluated the direct brewing of coffee with OP, preserving its natural composition within a beverage matrix. The observed increase in bioactive compounds, consistent with prior findings, reinforces the potential of OP as a functional ingredient. Given that OP is often considered an agro-industrial by-product, its incorporation into coffee formulations not only enhances the nutritional value, but also promotes sustainable food innovation. These findings set a new precedent for utilizing OP in functional beverages, emphasizing its potential for health-focused applications.

### 4.6. Sensory Evaluation of the C-OP 10% Brew

The incorporation of OP in functional products has been the subject of various studies that seek to take advantage of its nutritional benefits, such as the contribution of dietary fiber, which improves digestive health, and enrichment with natural antioxidants, such as hydroxytyrosol, oleuropein, and other compounds, to help reduce oxidative stress and prevent metabolic diseases [44,59,60].

In particular, the addition of 10% OP emerges as an optimal option, balancing chemical properties and sensory acceptance. A recent study evaluating OP incorporation in bread production found that higher concentrations of OP intensified bitterness and astringency, negatively affecting consumer acceptance of the product. These findings highlight the importance of optimizing OP levels to maximize health benefits while maintaining palatability in functional food formulations [44].

In another study conducted by Sucu et al. [59], they supplemented crossbred male lambs of Merino–Kıvırcık to evaluate the influence of two concentrations of OP, one at 10% and the other at 20%. In their work, there were no differences in most of the characteristics evaluated, such as fatty acid profile, polyphenol profile, and sensory profile, when using OP at 10 or 20% [59].

#### 4.6.1. Food Consumption in Murine Model of C-OP 10% Brew

The variation in food intake during the active phase of the experimental group provides valuable information on the impact of OP on feeding behavior. Although the analysis did not reveal effects between the groups, the fact that no consistent patterns of increase or decrease in food intake were observed during the evaluated period suggests that other factors, such as the individual behavior of the mice and palatability preferences (or specific taste) for a certain type of food, could be influencing the amount of food consumed [58,60].

#### 4.6.2. Body Weight Gain in Murine Model of C-OP 10% Brew

The inclusion of OP in the diet could be an effective strategy to improve metabolic health and reduce the risk of diseases related to weight gain in the population [44,50,59,60,61]. The inclusion has implications in the regulation of appetite and metabolism. The polyphenols present in OP have been associated with the improvement of insulin sensitivity and the reduction in inflammation, which may influence weight control and the prevention of obesity [62]; moreover, like Chlorogenic acid, these polyphenols have been shown to have properties that help to regulate glucose metabolism and body fat. According to a study by Vega et al. [63], Chlorogenic acids can reduce the absorption of carbohydrates in the intestine, which can help to control blood sugar levels and, therefore, weight gain [63].

The differences in body weight between the groups are not consistent over time. This could imply that other factors, such as individual variability in response to OP or metabolic adaptation to the diet, could be influencing the results [60]. It has been reported that the response to bioactive compounds can be modulated by genetic and environmental factors, which may result in variations in growth and development between individuals [59].

#### 4.6.3. Liquid Consumption in Murine Model of C-OP 10% Brew

In particular, the group supplemented with the C-OP 10% brew showed a 90% higher mean liquid intake than the control group, which could be related to the effects of the bioactive compounds present in the pomace, which can influence palatability and satiety [64].

The fact that fluid intake in the C-OP 10% brew group was higher on specific days, such as the third day of measurement, suggests that adaptation to OP could be affecting the consumption behavior over time. This pattern is consistent with that of previous studies that have documented that the introduction of new ingredients into the diet can temporarily alter fluid and food consumption habits [62]. However, the fact that there are no changes in liquid consumption, with or without C-OP 10% brew, indicates that the effect of the addition of OP may not be constant and could depend on other factors, such as individual variability in response to pomace or adaptation to the diet [65].

The consumption of polyphenols in the diet has become increasingly important worldwide, especially in the context of the Mediterranean diet [66]. Polyphenols, found in abundance in coffee, red wine, and especially in olive oil, have been associated with the prevention of chronic diseases such as T2DM and obesity [67]. Our study reveals that 552 mg of total polyphenols are provided in a 240 mL cup (the worldwide standard for coffee consumption) of C-OP 10% brew; therefore, our functional beverage would be providing approximately 46% of polyphenols out of a total of 1200 mg per day recommended [68,69].

In Spain, it has been estimated that the average daily intake of polyphenols is approximately 1365.1 mg, according to a study based on the Spanish National Dietary Intake Survey [70]; however, the intake of phenolic compounds varies according to the diet and eating habits of each population. A study conducted in Mexico estimated the average consumption of total polyphenols at 1.216 mg/day, with a median of 1.002 mg/day [71]. However, the exact amounts of phenols consumed globally are not clearly defined due to differences in diets and the lack of comprehensive studies in different populations.

Since our C-OP drink provides considerable amounts of polyphenols, its consumption could significantly contribute to covering the daily intake of polyphenols, being an alternative functional food also rich in antioxidants.

#### 4.6.4. Observed Behavior of Mice with and Without C-OP 10%

In the context of coffee consumption, caffeine, a primary active compound in coffee, has been shown to influence the circadian cycle, an internal biological process that regulates physiological and behavioral functions (like sleep–wake cycles, hormone release, and metabolism), in mammals [72].

In studies with murine models, it has been reported that coffee supplementation induces changes in behavior, particularly in motor activity and increasing active behavior. In the present work, the increase in active behavior observed in mice supplemented with the C-OP 10% brew could be attributed to the stimulation of the central nervous system by caffeine [73,74]. Throughout the experiment, it was observed that changes in activity were most evident in the first days of supplementation (day 1), suggesting an acute effect of caffeine. However, by days 7 and 14, this response seemed to be attenuated.

It is important to note that the C-OP 10% brew does not seem to enhance the effects of caffeine, but rather maintains its stimulation profile within the ranges expected for coffee consumption. These results suggest that the blend retains the cognitive and alertness benefits associated with caffeine without inducing overactivation of the central nervous system, which could favor its acceptance as a functional beverage.

### 4.7. Inhibitory Activity Against α-Amylase of C-OP 10%

At low concentrations (0.1–1 µL/mg), the brewed C-OP 10% brew presented 39 and 35.5% greater inhibition than the reference drug. This result suggests that the compounds present in OP and coffee may contribute to the regulation of blood glucose, the same as acarbose, but with an action profile that could be complementary [75]. This is consistent with the findings of Unuofin et al. [76], who indicated that plant extracts with a high polyphenol content can reduce the activity of α-amylase, suggesting that the combination of these ingredients could be beneficial for the management of T2DM.

Oyedemi et al. [77] found that phenolic compounds can inhibit the activity of α-amylase by covalently binding to the enzyme. This mechanism of action is similar to that of acarbose, suggesting that plant extracts containing flavonoids can be used as alternatives or complements to conventional treatments for T2DM [77].

Flavonoids have been shown to have activity against α-amylase, resulting in decreased starch digestion and, therefore, reduced glucose absorption [54,78]. Nunes et al. [62] found that flavonoids in OP, such as quercetin and rutin, can form complexes with proteins, which compromises the enzymatic activity of α-amylase.

## 5. Conclusions

As a by-product of the olive oil industry, OP is often considered waste, yet its use in functional beverages facilitates residue valorization, reduces environmental impact, and supports a circular economy. Rich in polyphenols and bioactive compounds, OP also offers potential metabolic health benefits. Its integration into food products aligns nutrition with sustainability, emphasizing its role in waste reduction and resource optimization in the food industry. The enrichment of coffee with OP presents a novel strategy to enhance its polyphenol content, improving antioxidant capacity and offering additional health benefits. This combination may have significant implications for the development of functional food products, particularly in the context of chronic diseases such as T2DM and obesity. However, the incorporation of OP may alter the sensory profile of coffee, potentially increasing bitterness or affecting consumer acceptance. Future studies should assess the sensory attributes and optimize formulations to balance health benefits with palatability, ensuring consumer acceptance. Addressing these factors will be essential to facilitating its integration into the food industry. These findings lay the groundwork for future research aimed at validating the efficacy of OP-enriched coffee in human populations and exploring its practical applications in dietary interventions. Further studies will be necessary to fully understand its long-term effects and potential as part of a broader health strategy.

## Figures and Tables

**Table 1:** Composition of C-OP brews with different coffee and OP concentrations.

Samples	Coffee (g)	OP (g)
Coffee	100	0
Olive pomace (OP)	0	100
C-OP 5%	95	5
C-OP 10%	90	10
C-OP 15%	85	15
C-OP 20%	80	20

**Table 2:** Compositions of coffee and OP brew extracts.

Brew	Soluble Solids	Total Phenols	Total Flavonoids	DPPH
	(g/100 mL)	mg CAE/mL	mg CE/mL	mmol TE/L
Coffee	1.20 ^a^ ± 0.20	1.00 ^a^ ± 0.10	3.60 ^NS^ ± 0.20	5.50 ^NS^ ± 1.00
OP	2.33 ^b^ ± 0.35	1.90 ^b^ ± 0.36	3.77 ^NS^ ± 0.61	5.40 ^NS^ ± 0.76

The values represent the means ± standard deviations (SD) of three determinations. Different letters in a column indicate statistical significance (*p* ≤ 0.05); NS: not significant differences, OP: olive pomace; DPPH: 2,2-diphenyl-1-picrylhydrazyl.

**Table 3:** Behavior observed in mice with consumption of C-OP 10% brew.

	Day	Control Group						C-OP 10% Brew Group					
		Dark (Active) Phase			Light (Inactive) Phase			Dark (Active) Phase			Light (Inactive) Phase		
Variable		1	7	14	1	7	14	1	7	14	1	7	14
Active behavior		++	++	++	+	+	+	++	+++	+++	+	++	++
Resting		++	++	++	+++	+++	+++	++	+	+	+++	++	++
Aggressiveness		Not observed						Not observed					
Anxiety													
Stereotypies													

Moderate = +, medium = ++, high = +++, C-OP = coffee and olive pomace.

## Data Availability

The original contributions presented in the study are included in the article, further inquiries can be directed to the corresponding author/s.

## Abbreviations

OP	Olive pomace
C-OP	Coffee and olive pomace
DPPH^•^	2,2-diphenyl-1-picrylhydrazyl
CAE	Chlorogenic acid equivalents
CE	(+)-catechin equivalents
AP	Active phase
IP	Inactive phase
T2DM	Type 2 diabetes mellitus
TE	Trolox equivalent
DNS	Dinitrosalicylic acid
